# Regulation of pancreatic stellate cell activation by Notch3

**DOI:** 10.1186/s12885-017-3957-2

**Published:** 2018-01-05

**Authors:** Haiyan Song, Yuxiang Zhang

**Affiliations:** Department of Biochemistry and Molecular Biology, Cancer Institute, Beijing Key Laboratory for Cancer Invasion and Metastasis Research, Capital Medical University, No. 10 Xitoutiao, You An Men, Fengtai District, Beijing, 100069 People’s Republic of China

**Keywords:** Pancreatic ductal adenocarcinoma, Pancreatic stellate cells, Notch3, Activation

## Abstract

**Background:**

Activated pancreatic stellate cells (PaSCs) are the key cellular source of cancer-associated fibroblasts in the pancreatic stroma of patients with pancreatic ductal adenocarcinoma (PDAC), however, the activation mechanism of PaSCs is not yet known. The Notch signaling pathway, components of which are expressed in stromal cells, is involved in the fibrosis of several organs, including the lung and liver. In the current study, we investigated whether Notch signal transduction is involved in PaSC activation in PDAC.

**Methods:**

The expression of Notch signaling pathway components in human PDAC was examined via immunohistochemical staining and assessed in mouse PaSCs using RT-qPCR and western blotting. Notch3 expression in both PDAC stromal cells and activated mouse PaSCs was evaluated using immunofluorescence, RT-qPCR and western blotting. The impact of siRNA-mediated Notch3 knockdown on PaSC activation was detected with RT-qPCR and western blotting, and the impact on PaSC proliferation and migration was detected using CCK-8 assays and scratch experiments. The effect of conditioned medium from PaSCs activated with Notch3 siRNA on pancreatic cancer (LTPA) cells was also detected with CCK-8 assays and scratch experiments. The data were analyzed for statistical significance using Student’s t-test.

**Results:**

Notch3 was overexpressed in both human PDAC stromal cells and activated mouse PaSCs, and Notch3 knockdown with Notch3 siRNA decreased the proliferation and migration of mouse PaSCs. The levels of markers related to PaSC activation, such as α-smooth muscle actin (α-SMA), collagen I and fibronectin, decreased in response to Notch3 knockdown, indicating that Notch3 plays an important role in PaSC activation. Furthermore, we confirmed that inhibition of PaSC activation via Notch3 siRNA reduced the proliferation and migration of PaSC-induced mouse pancreatic cancer (LTPA) cells.

**Conclusions:**

Notch3 inhibition in PaSCs can inhibit the activation, proliferation and migration of PaSCs and reduce the PaSC-induced pro-tumorigenic effect. Therefore, Notch3 silencing in PaSCs is a potential novel therapeutic option for patients with PDAC.

**Electronic supplementary material:**

The online version of this article (10.1186/s12885-017-3957-2) contains supplementary material, which is available to authorized users.

## Background

Pancreatic stellate cells (PaSCs) are myofibroblast-like cells found in exocrine areas of the pancreas, and they play an important role in the pathogenesis of pancreatitis and pancreatic cancer [1–3]. Fibrosis is a major feature of chronic pancreatitis and desmoplasia, a stromal reaction characteristic of pancreatic ductal carcinoma cancer (PDAC) [4]. In a normal pancreas, PaSCs constitute 4–7% of all pancreatic cells and are quiescent [5–7], however, PaSCs can switch between quiescent and activated phenotypes. In their quiescent state they have abundant vitamin-A-containing lipid droplets in their cytoplasm and express specific markers, such as desmin and glial fibrillary acidic protein (GFAP) [6]. When the pancreatic cells are injured, PaSCs transform into their active state, which is characterized by loss of the cytoplasmic vitamin-A-containing lipid droplets and upregulated expression of the cytoskeletal protein α-smooth muscle actin (α-SMA) [6, 7]. Activated PaSCs subsequently synthesize excessive extracellular matrix (ECM) proteins, such as collagen, fibronectin and laminin, and the proliferation and migration of PaSCs increases [8].

Recently, attention has been focused on the desmoplastic reaction in pancreatic cancer, specifically how it regulates cancer progression. This desmoplastic reaction occurs because activated PaSCs secrete large quantities of ECM proteins, including collagen types I, III, and IV, into the tumor microenvironment [7, 8]. There is strong evidence of a correlation between activated PaSCs and PDAC [9–11]. Thus, elucidation of the mechanism underlying PaSC transformation from a quiescent to an activated phenotype has many important implications.

Several signaling pathways and molecules that mediate PaSC activation have been identified, including Sonic hedgehog [12, 13], mitogen-activated protein kinases [14], peroxisome proliferator activated receptor γ [15, 16], the Janus kinase/signal transducer and activator of transcription pathway, and the transcription factor nuclear factor-kappa B [17–20].

However, more research is required to understand the details of PaSC activation. The Notch signaling family is an evolutionarily highly conserved signaling pathway. Notch activation plays critical roles in embryonic development, cell differentiation, cell proliferation and apoptosis [21, 22]. The canonical Notch signaling pathway is known as the CSL-dependent pathway. Notch receptor proteins can be activated by interacting with a family of ligands on adjacent cells. Upon activation, the Notch receptor is cleaved, and the intracellular domain of the Notch receptor (NICD) is released from the membrane into the cytoplasm and translocates into the nucleus. NICD in the nucleus binds with CSL (CBF1/Su(H)/LAG-1, also known as RBP-Jκ) and forms a transcriptional activation complex that acts as a potent transcriptional activator of CSL target genes, such as Hes1, and thus promotes downstream gene expression [23]. Notch signaling pathway components are highly expressed in PDAC [24–26], and inhibition of the Notch signaling pathway inhibits PDAC progression [27, 28]. These results indicate that the Notch signaling pathway plays an important role in PDAC occurrence and progression. In addition, the Notch pathway is involved in stromal cell activation during lung and hepatic fibrosis [29–32], however, the role of the Notch pathway in PaSC activation remains undefined. We hypothesized that components of the Notch pathway are present in PaSCs and that Notch signaling regulates the activation of these cells. To date, four Notch receptors have been identified in mammals, and the presence of multiple Notch receptors and ligands suggests that different receptors play different roles in PaSC activation. In the present study, we investigate the role of Notch signaling in PaSC activation.

## Methods

### Pancreatic tissues and animals

Human pancreatic cancer tissue microarrays were purchased from Xi’an Alena Biotechnology Co., Ltd. of China. For this study, male C57BL/6 J wild-type mice (6 weeks old, weight range 20–25 g) were supplied by the Laboratory Animal Services Center of Capital Medical University. Mouse LTPA cells (ATCC Number: CRL-2389™) were obtained from American Type Culture Collection (ATCC).

### Cell isolation and culture conditions

We isolated normal mouse PaSCs from the pancreas using the outgrowth method described by Apte and Bachem [5, 6]. PaSCs were cultured in DMEM/F12 (Gibco, New York, USA) containing 20% fetal bovine serum (FBS) (Gibco, New York, USA) and antibiotics (1% penicillin and streptomycin) (Beyotime, Haimen, China). PaSCs were confirmed by their fibroblast-like morphology and immunocytochemical positivity for PaSC markers such as α-SMA, collagen I and fibronectin.

### Immunohistochemical staining

Xylene and a graded alcohol series (ZSGB-BIO, Beijing, China) were used for dewaxing and rehydration. Subsequently, sections were treated with citrate salt buffer (pH 6.0) in the microwave for 15 min for antigen retrieval, followed by 3% hydrogen peroxide (ZSGB-BIO, Beijing, China) for 15 min to block endogenous peroxidase activity. Then, the samples were blocked with 5% donkey blood serum (Jackson, West Grove, USA) in phosphate-buffered saline (PBS) for 1 h at room temperature. The primary antibodies used in our experiments are listed in Table 1. The samples were then incubated with primary antibodies (against Notch1, Notch2, Notch3, Notch4, Jagged1, Jagged2, Delta1, Delta3 and Delta4) at 4 °C overnight, followed by incubation with secondary horseradish peroxidase (HRP)-conjugated antibodies (ZSGB-BIO, Beijing, China) for 1 h at room temperature. Next, diaminobenzidine (DAB) and hematoxylin (ZSGB-BIO, Beijing, China) were applied for staining and counterstaining. After dehydration with a graded alcohol series and xylene, the samples were sealed with coverslips and neutral gum.

**Table 1:** List of antibodies

	Host species	Dilution		Source/catalog no.
		IHC	WB	
Notch1	Rabbit	1:50	1:500	Santa Cruz/ sc-6014R
Notch2	Rabbit	1:500	1:2000	Lifespan/ LS-B399
Notch3	Rabbit	1:50	1:500	Santa Cruz/sc-5593
Notch4	Rabbit	1:50	1:500	Santa Cruz/sc-5594
HES1	Rabbit		1:200	Santa Cruz/ sc-25392
Jagged1	Rabbit	1:50		Santa Cruz/ sc-8303
Jagged2	Rabbit	1:50		Santa Cruz/ sc-5604
DLL1	Rabbit	1:50		Santa Cruz/ sc-9102
DLL3	goat	1:50		Santa Cruz/ sc-66513
DLL4	goat	1:50		Santa Cruz/ sc-18640
α-SMA	Mouse	1:100		Dako/M0851
α-SMA	Rabbit	1:100	1:1000	Abcam/ab5694
Fibronectin	Rabbit	1:50	1:1000	Proteintech/15613–1-AP
Collagen I	Rabbit	1:50	1:1000	Proteintech/14695–1-AP
CSL	Rabbit		1:1000	Cell Signaling/5313
GAPDH	Rabbit		1:10000	Sigma/G9545

### Immunofluorescence

Following dewaxing, rehydration and antigen retrieval, the samples were immunostained with mouse monoclonal anti-α-SMA (1:100) and rabbit polyclonal anti-Notch3 (1:50) antibodies at 4 °C overnight. The sections were then incubated with Alexa Fluor 594-conjugated donkey anti-rabbit IgG (Invitrogen, Chicago, USA) (1:1000) and Alexa Fluor 488-conjugated anti-mouse IgG (Invitrogen, Chicago, USA) (1:1000) for 1 h at room temperature. Nuclei were counterstained with 4′, 6-diamidino-2-phenylindole (DAPI; Sigma-Aldrich, Munich, Germany) for 5 min. The stained tissues were visualized using a laser scanning confocal microscope (Olympus, Postfach, Hamburg, Germany).

### Immunocytochemical staining

Mouse PaSCs from adherent cultures were digested with 0.25% trypsin/EDTA and centrifuged at 800 rpm for 3 min. The cell pellets were resuspended in complete medium. After preparing 6-well plates with coverslips, cell suspension was added into each well. The cells were cultured at 37 °C in 5% CO_2_ for 48 h, washed with PBS and fixed with 4% paraformaldehyde (PFA) (ZSGB-BIO, Beijing, China) for 15 min. Then, cells were treated with 10% donkey serum at room temperature for 1 h and incubated with the following primary antibodies: mouse monoclonal anti-α-SMA (1:100), rabbit polyclonal anti-fibronectin (1:50), and rabbit polyclonal anti-collagen I (1:50) at 4 °C overnight. The cells were then incubated with Alexa 594-conjugated anti-rabbit IgG (Invitrogen, Chicago, USA) (1:1000) or Alexa 488-conjugated anti-mouse IgG (Invitrogen, Chicago, USA) (1:1000) for 1 h at room temperature. Nuclear staining was performed with 4′,6-diamidino-2-phenylindole (DAPI; Sigma-Aldrich Munich, Germany) for 5 min. The stained coverslips were visualized using a Nikon 80i fluorescence microscope.

### siRNA-mediated Notch3 knockdown in PaSCs

One Notch3 siRNA (sc-37,136) was purchased from Santa Cruz and another was synthesized by Shanghai Genepharma Co. Ltd. (Shanghai, China). The Notch3 siRNA sequence was (5′-3’)GCCAGAACUGUGAAGUCAATT, and the control siRNA sequence was (5′-3′) UUCUCCGAACGUGUCACGUTT. PaSCs transfection was performed using the following steps. PaSCs were seeded into 6-well plates and transfected with Notch3 siRNA (50 nM) or negative siRNA using Lipofectamine 2000. After 48 h of transfection, mRNA and protein were extracted from the cells. Quantitative real-time reverse transcription polymerase chain reaction (RT-qPCR) and western blotting were used to confirm the Notch3 knockdown efficiency.

### Cell proliferation

Cells (2 × 10^5^) were seeded in a 24-well plate in 800 μl of DMEM/F12 containing 20% FBS and incubated at 37 °C in 5% CO_2_ for 24 h. Mouse PaSCs in serum-free medium were transfected with Notch3 siRNA using Lipofectamine 2000 transfection reagent (Invitrogen, Chicago, USA) in accordance with the manufacturer’s instructions. Five h after transfection, serum-free medium was replaced with complete medium. At 24, 48 and 72 h after transfection, cell growth was measured using a CCK-8 cell viability assay (AAT Bioquest, USA) according to the manufacturer’s instructions.

To study the effect of PaSCs on mouse pancreatic cancer cells (LTPA cells), conditioned medium from mouse PaSCs was collected. Mouse PaSCs were seeded into a 6-well plate in complete medium for 24 h and then transfected with Notch3 siRNA. Forty-eight hours after transfection, the medium conditioned by PaSCs was collected. LTPA cells were incubated in the PaSC-conditioned medium for 24, 48 and 72 h, and their growth was measured using the CCK-8 cell viability assay (AAT Bioquest, USA) according to the manufacturer’s instructions.

### Cell migration assay

Mouse PaSCs were seeded in a 6-well plate (2 × 10^5^ cells) and incubated for 24 h. A scratch was made using a 1 ml pipette tip before the cells were transfected with Notch3 siRNA. Images were captured at 0, 24 and 48 h after transfection under an inverted microscope. ImageJ software was used to calculate the area of the scratch. Then, the percentage of wound closure was calculated and compared with that of the negative control.

To study the effect of PaSCs on mouse LTPA tumor cell migration, conditioned medium from mouse PaSCs was collected as described above. Mouse LTPA cells were seeded into the top of transwell chambers at 3 × 10^5^ cells/ml. The bottom of the transwell chambers contained 600 μl of PaSC-conditioned medium. After 24 h, LTPA cells in the top chambers were swabbed away with a Q-tip. The membranes were washed three times with PBS and then fixed with 4% PFA for 20 min and with 0.1% crystal violet (Sigma-Aldrich, Munich, Germany) for 15 min. LTPA cells were counted in at least in five random fields and photographed via microscopy (×200).

### RT-qPCR

Total RNA was isolated from non-activated and activated mouse PaSCs 48 h after transfection with either Notch3 siRNA or control siRNA using TRIzol reagent (Invitrogen, Chicago, USA). The RNA concentration was measured using a NanoDrop® ND-1000 Spectrophotometer (Wilmington, DE). cDNA was synthesized with a RevertAid first strand cDNA synthesis kit (k1622, Thermo Scientific, Waltham, USA). RT-qPCR primers were synthesized by Sangong Biotech (Shanghai) and are listed in Table 2. RT-qPCR was conducted using a Mx3000p RT-PCR detection system and TransStart Top Green qPCR SuperMix (AQ131–02, Transgen Biotech, Beijing, China). The relative gene expression levels were normalized to glyceraldehyde 3-phosphate dehydrogenase (GAPDH) levels.

**Table 2:** Primers used for RT-qPCR

Primer	Forward sequence 5′-3’	Reverse sequence 5′-3’
Notch1	TCGTGCTCCTGTTCTTTGTG	CTCTCCGCTTCTTCTTGCTG
Notch2	GCAGGAGCAGGAGGTGATAG	ATGAGAAGCCAGGAGAGCAG
Notch3	TGGCTATGCTGGTGACAGTT	AGGGGGACAGGAACAGAGAT
Notch4	AATGCCAAGGTCAGGAACAC	AGCCCTCATCACACACACAC
α-SMA	AATGGCTCTGGGCTCTGTAA	CTCTTGCTCTGGGCTTCATC
Fibronectin	GAAGTCGCAAGGAAACAAGC	GTAGGTGAACGGGAGGACAC
CollagenI	TGACTGGAAGAGCGGAGAGT	GACGGCTGAGTAGGGAACAC
HES1	GGCGAAGGGCAAGAATAAAT	TGCTTCACAGTCATTTCCAGA
GAPDH	GGTTGTCTCCTGCGACTTCA	TGGTCCAGGGTTTCTTACTCC

### Western blotting

Proteins were isolated from non-activated and activated mouse PaSCs 48 h after transfection with either Notch3 siRNA or control siRNA using radioimmunoprecipitation assay buffer (Beyotime Bio, Haimen, China) containing a protease inhibitor cocktail and PMSF. Cells were centrifuged at 12,000 g for 30 min, and supernatant fractions were collected. Protein was measured with a Pierce™ BCA protein assay kit according to the manufacturer’s instructions (Prod #23225, Thermo Scientific, Waltham, USA). Equal amounts of protein were loaded and separated on 8% or 10% PAGE gels and transferred onto nitrocellulose filter membranes (Millipore, Darmstadt, Germany). The membranes were incubated with 5% milk for 1 h at room temperature and then probed with primary antibodies overnight at 4 °C. The primary antibodies are shown in Table 1. Subsequently, the membranes were incubated with peroxidase-conjugated AffiniPure goat anti-rabbit IgG (H + L) (ZB-2301, ZSGB-Bio) for 1 h at room temperature. The proteins were detected with enhanced chemiluminescence (Millipore, Darmstadt, Germany) using an LAS3000 System (Fujifilm, Japan). Protein levels were normalized to GAPDH levels and quantified using ImageJ software (NIH).

### Statistical analysis

The data are presented as the mean ± SD. Comparisons between two groups were analyzed with a two-sided Student’s t-test using SPSS16.0 software. *P* < 0.05 was considered statistically significant. All experiments were repeated three to six times.

## Results

### Expression of notch receptors and ligands in human PDAC stroma

**Fig. 1:**
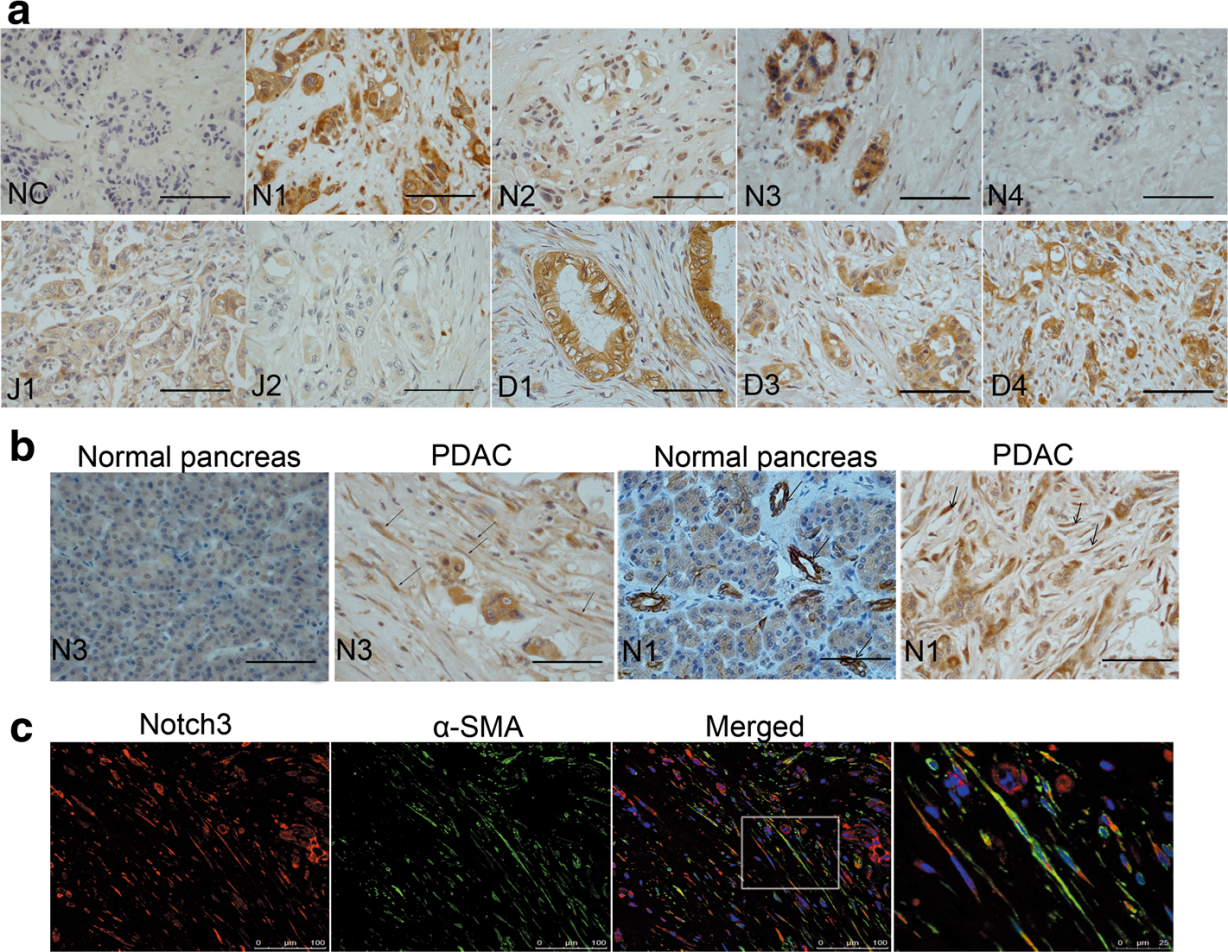
Expression of Notch1-Notch4 and the Notch ligands Jagged1, Jagged2, DLL1, DLL3, and DLL4 in PDAC. **a** Representative immunohistochemistry images of Notch1-Notch4 receptors (N1,N2,N3,N4) and Jagged1 (J1), Jagged2 (J2), DLL1 (D1), DLL3 (D3), and DLL4 (D4) ligands in PDAC. NC: negative control. **b** Representative immunohistochemistry images of Notch1 and Notch3 protein expression in PDAC and in normal pancreas. **c** Representative double immunofluorescence staining of α-SMA (green) and Notch3 (red) in PDAC stroma. High magnification image is shown on the right. Scale bars: 50 μm in (**a**) and (**b**)

To investigate the expression of Notch signaling components in human PDAC, we performed an immunohistochemistry (IHC) analysis. We found that both Notch receptors and ligands were expressed in PDAC tumor cells, but the degree of expression varied. Notch1 and Notch3 and the Notch ligands DLL1, DLL3 and DLL4 were highly expressed, and Notch2 and Notch4 and the Notch ligands Jagged1 and Jagged2 were slightly expressed (Fig. 1a). We also identified Notch3 and Notch1 expression in PDAC stroma (Fig. 1b). The results of immunofluorescence co-localization demonstrated that Notch3 was expressed in α-SMA-positive activated PaSCs (Fig. 1c).

### Primary culture and identification of non-activated and activated mouse PaSCs

**Fig. 2:**
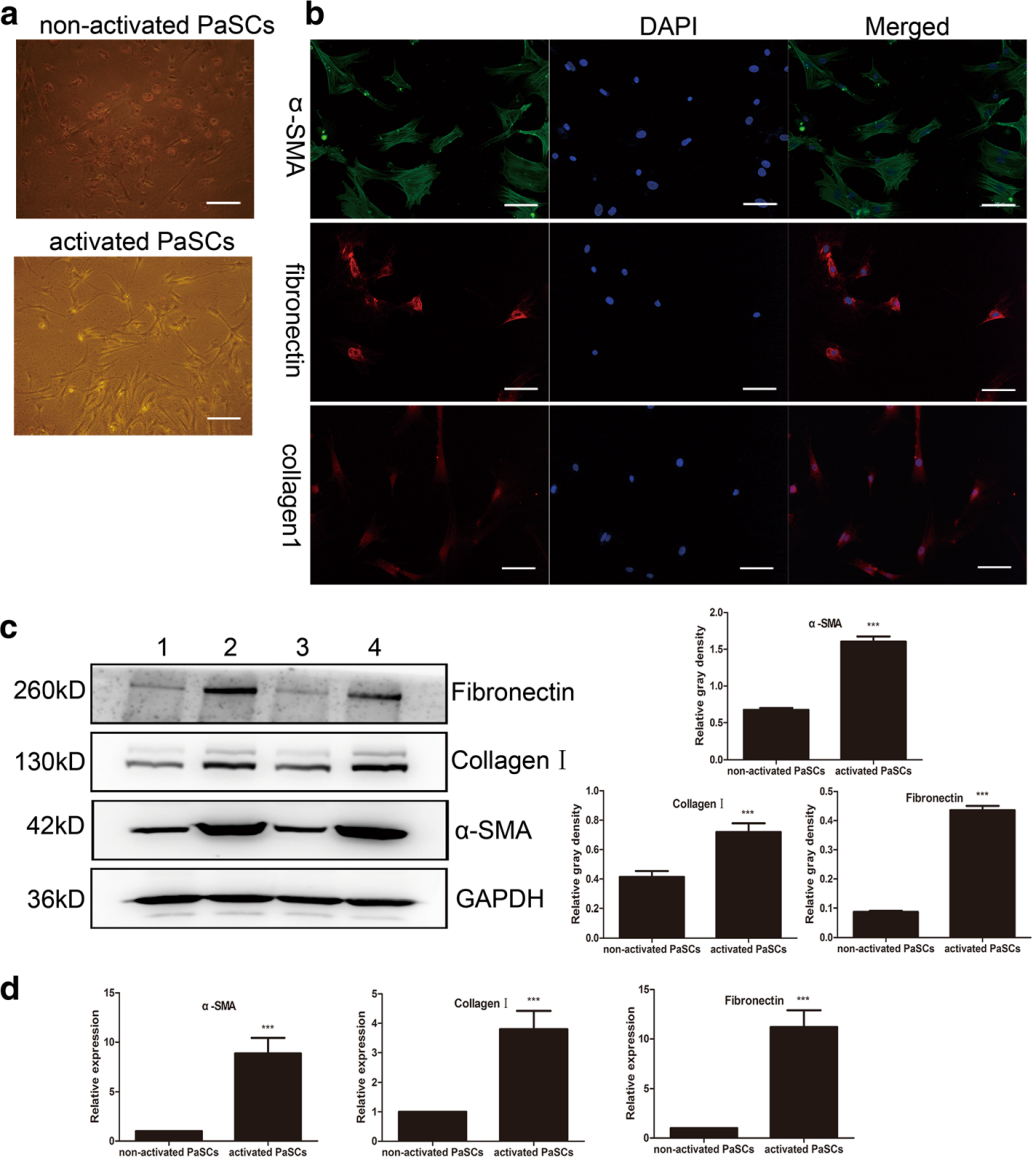
Culture and identification of primary mouse PaSCs. **a** Representative oil red O staining in mouse non-activated and activated PaSCs. **b** Immunofluorescence staining of α-SMA, collagen I and fibronectin in mouse PaSCs. Nuclei were counterstained with DAPI. **c** Representative western blotting images showing the α-SMA, collagen I and fibronectin expression in non-activated and activated PaSCs; densitometry analyses of the blots are also shown (groups 1 and 3 represent non-activated PaSCs; groups 2 and 4 represent activated PaSCs). **d** Representative RT-qPCR results showing the α-SMA, collagen I and fibronectin mRNA expression in non-activated and activated PaSCs. Scale bars: 50 μm in (**a**) and (**b**). The data are presented as the mean ± SD. ***P < 0.001; *n* = 4; (t-test); Student’s t-test

According to the literature, PaSCs in the normal pancreas have similar function compared to PaSCs in PDAC, such as promoting tumor cell growth and metastasis [33, 34]. We used primary normal mouse PaSCs as a model to study the possible activation mechanism of PaSC, and we used oil red O staining [33] to identify non-activated and activated PaSCs. We identified numerous lipid droplets in early-passage primary cells, indicating that these cells were non-activated PaSCs (Fig. 2a). After growth on a plastic surface for 5 days, the lipid droplets in these primary cells disappeared, and the morphology of the cells changed, the cells became flattened and developed long cytoplasmic extensions, which are characteristics of activated PaSCs (Fig. 2a). The transition of quiescent PaSCs to an activated myofibroblastic phenotype was accompanied by changes in the cytoskeleton.

The activated PaSCs were positive for α-SMA, collagen I and fibronectin (Fig. 2b). Quantitative reverse transcription polymerase chain reaction (RT-qPCR) and sodium dodecyl sulfate polyacrylamide gel electrophoresis (SDS-PAGE) followed by western blotting were used to detect α-SMA, collagen I and fibronectin mRNA and protein, respectively. Densitometry analyses revealed that compared to non-activated PaSCs activated PaSCs had higher mRNA and protein levels of α-SMA (1.000 ± 0 vs 8.854 ± 5.485 and 0.6738 ± 0.0668 vs 1.604 ± 0.1725, respectively), collagen I (1.000 ± 0 vs 3.803 ± 2.154 and 0.4138 ± 0.09837 vs 0.7192 ± 0.1449, respectively) and fibronectin (1.000 ± 0 vs 11.20 ± 5.890 and 0.08677 ± 0.00979 vs 0.4358 ± 0.0366, respectively) (*n* = 4, *P* < 0.001; Fig. 2c-d). Taken together, these results indicate that we cultivated PaSCs.

### The expression of notch signaling pathway components in non-activated and activated PaSCs

**Fig. 3:**
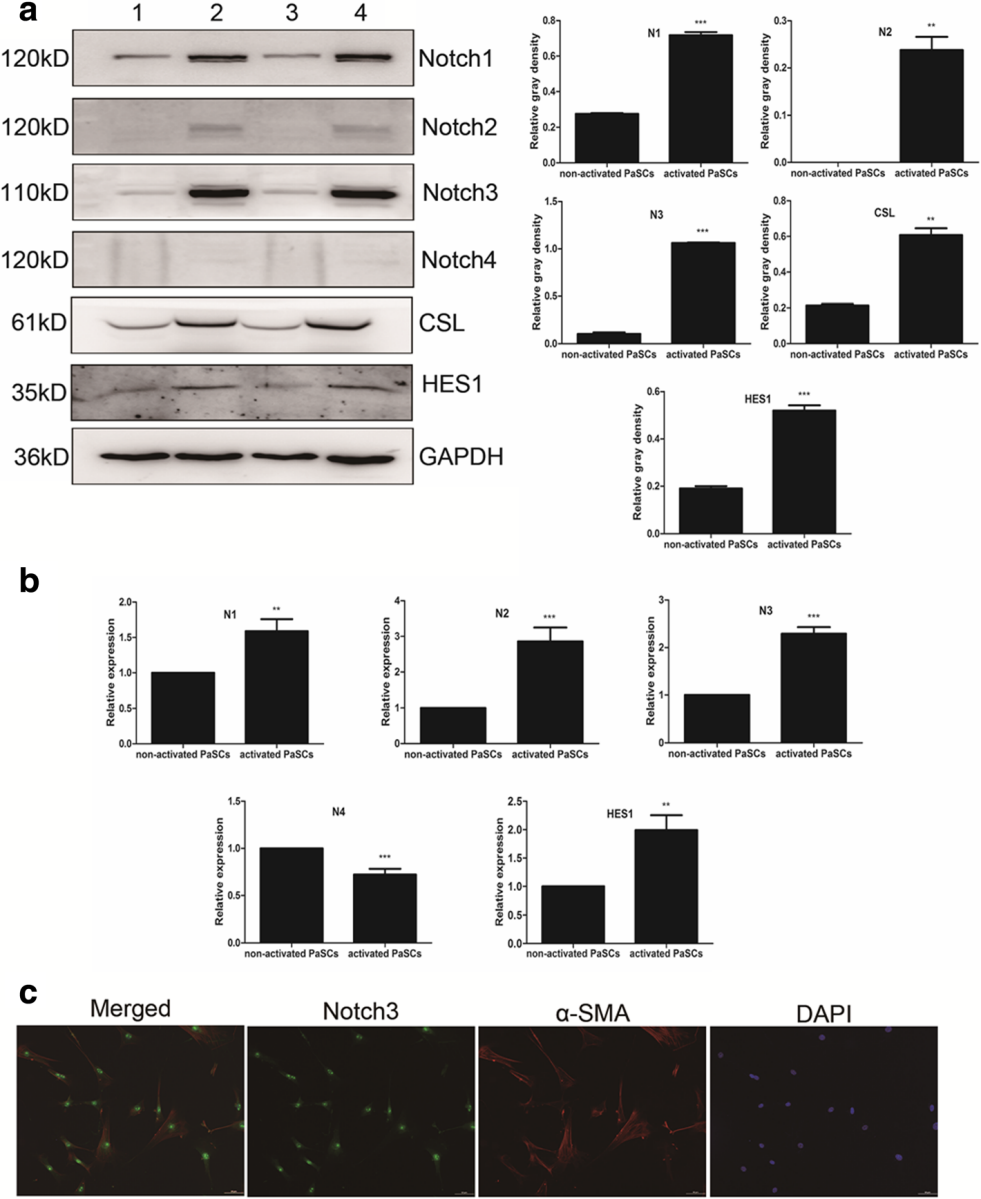
Notch receptor expression in primary mouse PaSCs. **a** Representative western blotting images showing the Notch1–4, CSL and HES1 protein expression in non-activated and activated PaSCs; densitometry analyses of the blots are also shown (groups 1 and 3 represent non-activated PaSCs; groups 2 and 4 represent activated PaSCs). **b** RT-qPCR results showing the Notch1–4 and HES1 mRNA expression in non-activated and activated PaSCs. **c** Representative double immunofluorescence staining of α-SMA (red) and Notch3 (green) in primary mouse PaSCs. Scale bars: 50 μm in (**c**). The data are presented as the mean ± SD, **P < 0.01 and ***P < 0.001; *n* = 4; Student’s t-test

Western blotting and RT-qPCR were used to detect changes in the expression of Notch family proteins induced by PaSCs activation. Levels of Notch1 (0.7169 ± 0.03594 vs 0.2761 ± 0.008455, *P* < 0.001), Notch2 (0.2378 ± 0.05646 vs 0, *P* < 0.01), Notch3 (1.061 ± 0.01039 vs 0.1033 ± 0.03333, *P* < 0.001), the transcription factor CSL (0.6074 ± 0.07683 vs 0.2139 ± 0.01509, *P* < 0.01) and the Notch target gene HES1 (0.4100 ± 0.02194 vs 0.2035 ± 0.004786, *P* < 0.001) were upregulated in activated PaSCs compared to non-activated PaSCs (*n* = 4; Fig. 3a). Notch4 was not detectable (Fig. 3a). The mRNA levels of Notch1 (1.587 ± 0.5973 vs 1.000 ± 0, *P* < 0.01), Notch2 (2.858 ± 1.352 vs 1.000 ± 0, *P* < 0.001), Notch3 (2.291 ± 0.4797 vs 1.000 ± 0, *P* < 0.001) and HES1 (1.992 ± 0.9125 vs 1.000 ± 0, *P* < 0.001) were upregulated in activated PaSCs compared to non-activated PaSCs, respectively (*n* = 4; Fig. 3b), which is consistent with the trend in protein levels. In addition, Notch4 mRNA was downregulated (0.7216 ± 0.2144 vs 1.000 ± 0, *P* < 0.001) (*n* = 4). Using immunofluorescence double-staining, we demonstrated that the Notch3 protein was highly expressed in α-SMA-positive but not α-SMA-negative PaSCs (Fig. 3c). Taken together, these results demonstrate Notch3 expression in activated PaSCs.

### Effect of Notch3 inhibition on PaSC activation

**Fig. 4:**
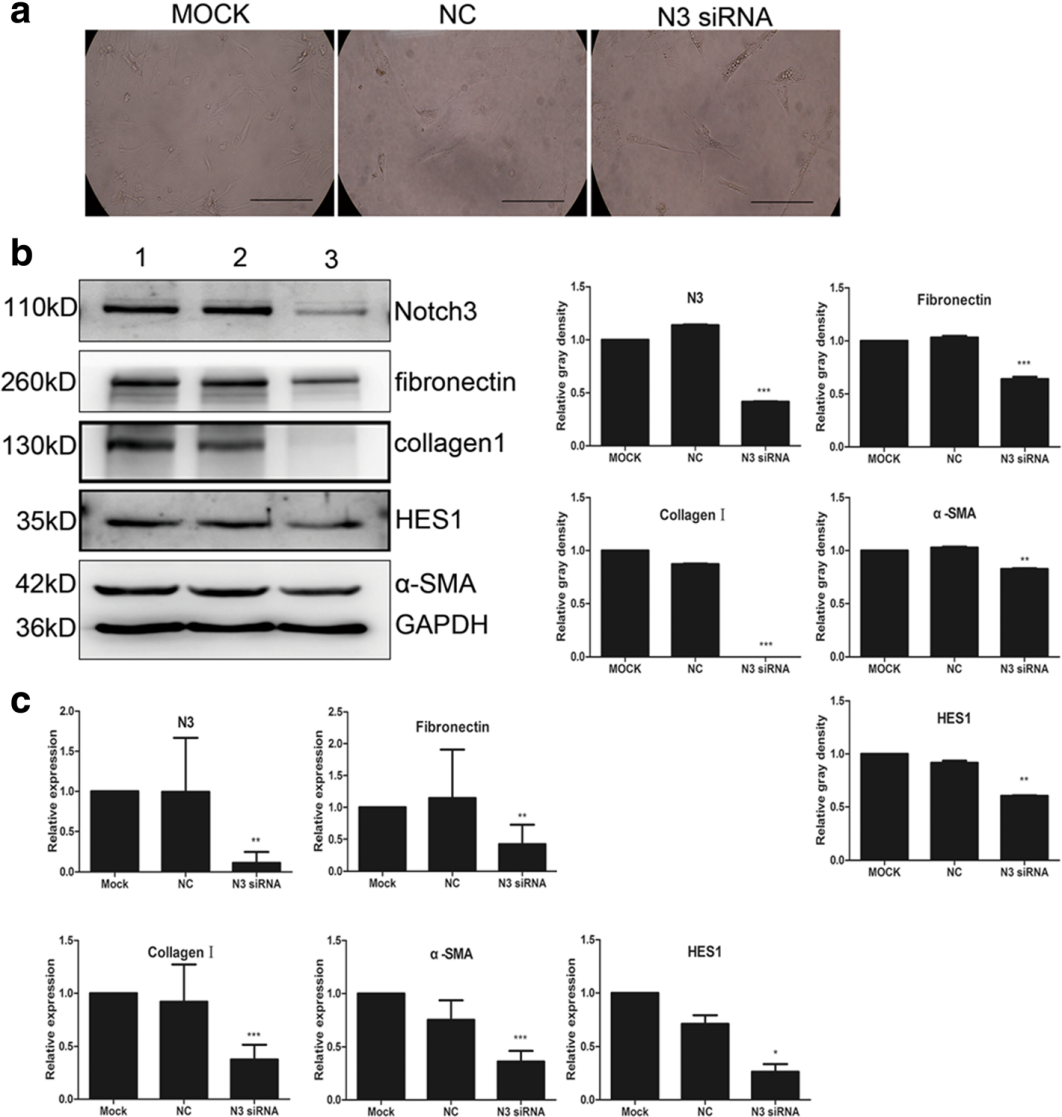
Effect of siRNA-mediated Notch3 inhibition on mouse PaSC activation. **a** Transfection of Notch 3 siRNA in mouse PaSCs activation after 48 h, the morphological changes in PaSCs. **b** Representative western blotting images showing the effect of siRNA-mediated Notch3 inhibition on PaSC activation markers, such as α-SMA, fibronectin and collagen I, and on the Notch target gene HES1; densitometry analyses of the blots are also shown. **c** RT-qPCR results showing the effect of siRNA-mediated Notch3 inhibition on PaSC activation markers, such as α-SMA, fibronectin and collagen I, and on the Notch target gene HES1 at the transcriptional level. Scale bars: 100 μm in (**a**). The data are presented as the mean ± SD, *P < 0.05, **P < 0.01, and ***P < 0.001; *n* = 4; Student’s t-test

To investigate whether Notch3 downregulation inhibits mouse PaSC activation, Notch3 siRNA (50 nM) was used to knock down Notch3 expression. Cytoplasmic lipid droplets reappeared in activated PaSCs 48 h after transfection with Notch3 siRNA (Fig. 4a). We observed a significant downregulation of Notch3 protein in cells transfected with Notch3 siRNA but not in those transfected with control siRNA (0.1117 ± 0.1368 vs 0.9938 ± 0.6741; *P* < 0.01; *n* = 4) (Fig. 4b). α-SMA, collagen I and fibronectin are markers of activated PaSCs, and α-SMA, collagen I and fibronectin were all significantly lower in PaSCs treated with Notch3-specific siRNA. In control-siRNA-treated cells, α-SMA, collagen I and fibronectin levels were 1.028 ± 0.01647, 0.8719 ± 0.007824 and 1.032 ± 0.02623, respectively, and in Notch-3-siRNA-treated cells, these levels were reduced to 0.8252 ± 0.01324 (*P* < 0.001), 0.0000 (*P* < 0.001) and 0.6397 ± 0.03654 (*P* < 0.01), respectively (Fig. 4b). The protein expression level of the Notch target gene HES1 was also downregulated by Notch3 siRNA (0.9155 ± 0.03396 vs 0.6038 ± 0.01053, *P* < 0.01; Fig. 4b).

We also confirmed downregulation of α-SMA, collagen I and fibronectin mRNA in PaSCs treated with Notch3-specific siRNA. The mRNA levels of α-SMA, collagen I and fibronectin fell from 0.7513 ± 0.1846, 0.9198 ± 0.3538, and 1.145 ± 0.7626 in cells treated with control siRNA to 0.3620 ± 0.09951 (*P* < 0.001), 0.3762 ± 0.1392 (*P* < 0.001), and 0.4260 ± 0.2995 (*P* < 0.01), respectively, in cells treated with Notch3 siRNA (Fig. 4c). The mRNA of the Notch target gene HES1 was significantly downregulated in PaSCs transfected with Notch3 siRNA compared to PaSCs transfected with control siRNA (0.2637 ± 0.1776 vs 0.7092 ± 0.1991, *P* < 0.05, *n* = 4; Fig. 4c).

### PaSCs are activated by culture in conditioned medium from PDAC cells

The activation of PaSCs by treatment with mouse PDAC tumor cell (LTPA cell)-conditioned medium (2 ml) was assessed by analyzing the expression of markers of activated PaSCs. After 3 days of standard culture, PaSCs were further cultured with LTPA-conditioned medium for 24 h and then transfected with either Notch3 siRNA or control siRNA for 48 h to determine if Notch3 siRNA suppressed the PaSC activation induced by LTPA-conditioned medium. We found that Notch3-specific siRNA downregulated the expression of PaSC activation markers (Additional file 1: Figure S1). These results collectively demonstrate that Notch3 plays an important role in the transition of PaSCs from a quiescent to an activated state.

### Effect of Notch3 siRNA on migration and proliferation of PaSCs

**Fig. 5:**
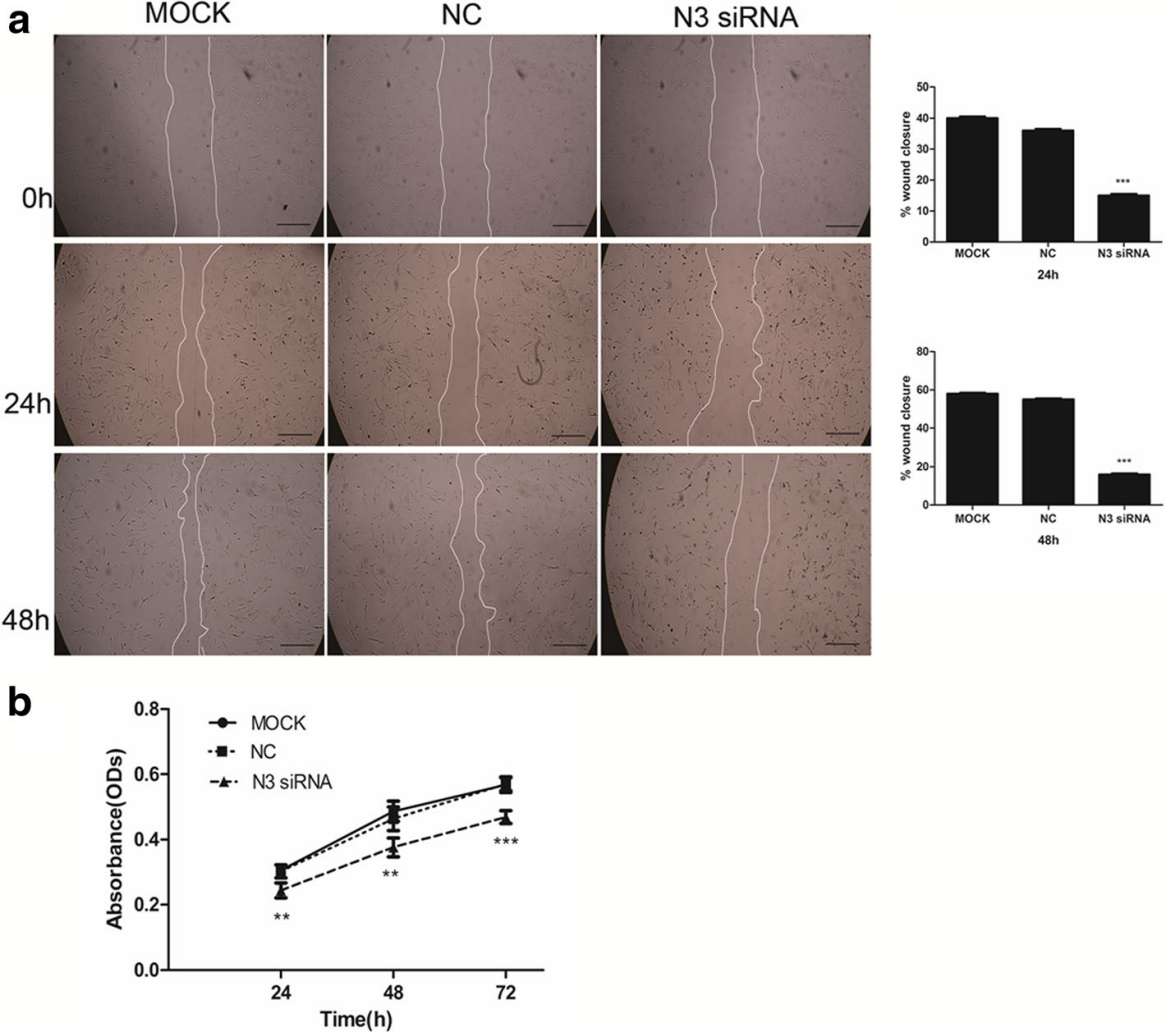
Effect of Notch3 siRNA on migration and proliferation of mouse PaSCs. **a** Representative microscopic images showing the effect of Notch3 siRNA on the migration of mouse PaSCs; the semi-quantitative image analysis is also presented (*n* = 4). **b** Cell growth curve showing that transfection of mouse PaSCs with Notch3 siRNA significantly reduced PaSC proliferation compared to negative control siRNA. Scale bars: 100 μm in (**a**). The data are presented as the mean ± SD, **P < 0.01 and ***P < 0.001; *n* = 6; Student’s t-test

We examined whether Notch3 plays a role in the migration and proliferation of PaSCs. We used a scratch assay (wound healing assay) and a cholecystokinin-8 (CCK-8) assay to measure the effect of Notch3 siRNA on migration and proliferation, respectively, of mouse PaSCs (see Methods). The scratch assay showed that Notch3 siRNA (50 nM) inhibited wound closure (scratch gap), and therefore migration of PaSCs, compared to control siRNA. As shown in Fig. 5a, mock-control PaSCs (non-transfected) and control-siRNA-treated PaSCs migrated into the gap formed by the scratch made in the cell monolayer and covered 40.25% and 36.44% of the gap surface area 24 h after transfection and 58% and 55.07% 48 h after transfection, respectively. In contrast, PaSCs transfected with Notch3 siRNA migrated much more slowly than both mock-control-treated and siRNA-control-treated PaSCs, filling only 15.48% and 18.02% of the gap at 24 h and 48 h, respectively (Notch3 siRNA-treated PaSCs vs control-siRNA-treated PaSCs at 24 h: 15.48 ± 0.9891 vs 36.44 ± 0.7617, *P* < 0.001; and at 48 h: 18.02 ± 1.340 vs 55.07 ± 1.441, *P* < 0.001; *n* = 4).

These results indicate that Notch3 knockdown severely inhibits the migratory activity of PaSCs.

We further investigated whether downregulation of Notch3 by siRNA inhibited PaSC proliferation and found that Notch3 knockdown significantly inhibited PaSC proliferation (Fig. 5b) (24 h: 0.2440 ± 0.02298 vs 0.3022 ± 0.02005, *P* < 0.01; 48 h: 0.3838 ± 0.03224 vs 0.4732 ± 0.03949, *P* < 0.01; 72 h: 0.4692 ± 0.01975 vs 0.5704 ± 0.01991, *P* < 0.001; *n* = 6). These data suggest that Notch3 regulates PaSC migration and proliferation.

### Effect of PaSC-conditioned medium on migration and proliferation of tumor cells

**Fig. 6:**
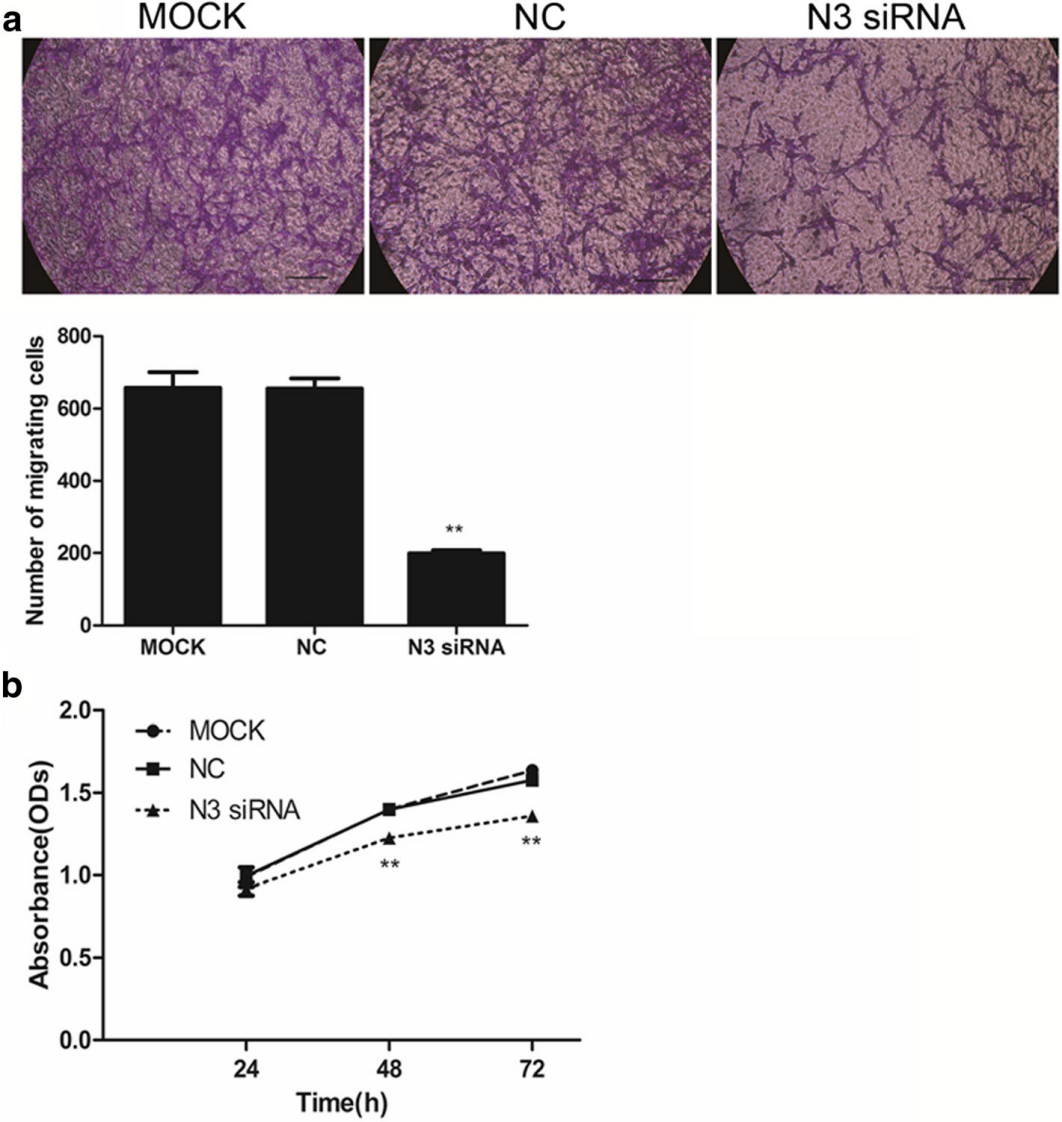
Notch3 siRNA-mediated effects of PaSCs on migration and proliferation of LTPA cells. **a** The number of migratory LTPA cells after incubation with conditioned medium obtained from PaSCs transfected with Notch3 siRNA was significantly reduced compared with that of the negative control cells; the semi-quantitative image analysis is also shown (n = 4). **b** LTPA cell growth curves after incubation with conditioned medium obtained from PaSCs transfected with Notch3 siRNA showing significantly reduced LTPA proliferation compared to that of negative control cells. Scale bars: 100 μm in (**a**). The data are presented as the mean ± SD. **P < 0.01; *n* = 6; Student’s t-test

Control-siRNA-treated and Notch3-siRNA-treated PaSCs were cultured for 48 h, and then, the culture medium was collected and used to culture LTPA (mouse PDAC) cells. The migration and proliferation of LTPA cells were then examined. Transwell experiment results showed that the migration of LTPA cells cultured in the conditioned medium from Notch3 siRNA-treated PaSCs was significantly reduced compared with that of LTPA cells cultured in conditioned medium from the control-siRNA-transfected PaSCs (Fig. 6a; 199.3 ± 14.05 vs 654.7 ± 49.14, *P* < 0.01; *n* = 4). We also used CCK-8 assays to determine the effect of PaSC-conditioned medium on LTPA cell proliferation. We observed that the proliferation of LTPA cells cultured with conditioned medium from Notch3-siRNA-transfected PaSCs was decreased compared with that of the LTPA cells cultured with conditioned medium from control-siRNA-transfected PaSCs (Fig. 6b; 48 h: 1.234 ± 0.03753 vs 1.422 ± 0.08884, *P* < 0.01; 72 h: 1.359 ± 0.03249 vs 1.577 ± 0.07606, *P* < 0.01; *n* = 6). These data indicate that inhibition of PaSC activation by Notch3 siRNA reduces tumor cell migration and proliferation, presumably by releasing currently unidentified factors into the medium.

## Discussion

One of the features of PDAC is the presence of extensive desmoplasia. The desmoplastic stroma consists of ECM and stromal cells [35]. PaSCs are the most numerous stromal cells and are responsible for ECM production. Thus, they play an important role in regulating the PDAC tumor microenvironment [2, 36, 37]. In a healthy pancreas, PaSCs remain in a quiescent state, exhibit abundant lipid droplets rich in vitamin A in their cytoplasm [1], and express desmin and glial fibrillary acidic protein (GFAP) [6]. However, when the pancreas is injured by either inflammation or tumor growth, the PaSCs are activated by growth factors, cytokines or oxidative stress [38]. Activated PaSCs transdifferentiate into myofibroblast-like cells, express the fibroblast activation marker α-SMA, acquire proliferative capacity, and increase the synthesis of collagen and fibronectin [7]. Although a number of studies have shown that growth factors (such as platelet-derived growth factor (PDGF) and transforming growth factor (TGF-β1), cytokines (such as interleukin-6, interleukin-8 and tumor necrosis factor (TNF-α) and oxidative stress products activate PaSCs [39–43], the activation mechanism is not yet fully understood.

Recently, Notch1 has been shown to be involved in myofibroblast activation and to regulate α-SMA expression in lung fibrosis [32]. In addition, the Notch3 receptor plays a critical role in the transition of quiescent hepatic stellate cells (HSCs) into myofibroblastic HSCs in hepatic fibrosis [29–31]. In the present study, we found that Notch3 was highly expressed in α-SMA-positive cells in human pancreatic tumor tissue but not in normal pancreatic cells, suggesting that Notch3 participates in PaSC activation.

Quiescent PaSCs can be activated when PaSCs in normal pancreatic tissue are cultured in vitro*.* Although gene microarray analysis has shown gene expression differences between cultured cancer-associated PaSCs and normal PaSCs, the cells exert the same effects on pancreatic cancer cells [34]. Primary PaSCs isolated from normal pancreatic specimens are qualitatively indistinguishable from pancreatitis- and pancreatic cancer-derived PaSCs [33]. Furthermore, immortalized PaSCs have the same response to TGF-β1 and PDGF as their cultured primary cell counterparts [44, 45]. In the present study, we investigated the role of Notch signaling in PaSC activation using primary cultured PaSCs from normal mouse pancreas.

We observed that Notch3 is highly expressed in activated PaSCs, but not in non-activated PaSCs. Moreover, the levels of PaSC markers, such as α-SMA, collagen I and fibronectin were reduced by knocking down Notch3 expression in PaSCs. This suggests that Notch3 plays a crucial role in PaSC activation. In addition, we showed that Notch3 knockdown reduced migration and proliferation of PaSCs, which are required for the formation of desmoplasia [46]. We also found that conditioned medium from cultures of activated PaSCs enhanced the proliferation of LTPA PDAC cells. Thus, Notch3 is a potential target for inhibition of PaSC activation and thus desmoplasia.

## Conclusions

In summary, we have demonstrated for the first time that Notch3 plays an important role in PaSC activation, migration and proliferation, and thus, the canonical Notch signaling pathway is involved in desmoplastic stroma formation in PDAC.

## Additional file

Additional file 1: Figure S1. Representative western blotting images showing α-SMA, collagen I and fibronectin expression in PaSCs; densitometry analyses of the blots is also shown. 1. MOCK; 2. NC; 3. Notch3 siRNA; 4. LTPA-conditioned medium; 5. LTPA-conditioned medium + Notch3 siRNA. **P* < 0.05, ***P* < 0.01, and ****P* < 0.001; Student’s t-test; *n* = 4. Bars represent mean ± SD. (TIFF 749 kb)

## Abbreviations

CP: Chronic pancreatitis
DAB: Diaminobenzidine
ECM: Extracellular matrix
GFAP: Glial fibrillary acidic protein
MAPK: Mitogen-activated protein kinase
PaSCs: Pancreatic stellate cells.
PBS: Phosphate-buffered saline.
PDAC: Pancreatic ductal adenocarcinoma.
α-SMA: α-smooth muscle actin.
