# Supplementary figures and images for: Regulation of pancreatic stellate cell activation by Notch3
Source: BMC Cancer. 2018 Jan 5;18:36. doi: 10.1186/s12885-017-3957-2 (PMC5756326; doi:10.1186/s12885-017-3957-2)

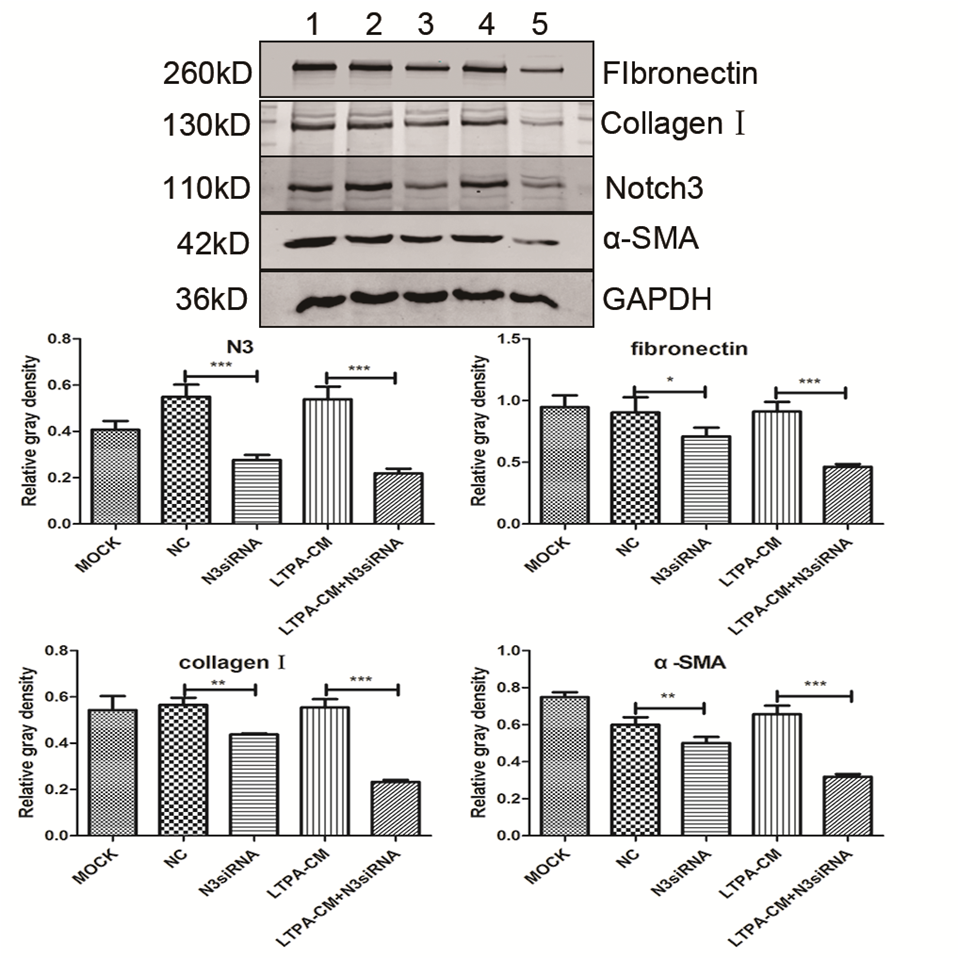

Supplement: Additional file 1: Figure S1. — Representative western blotting images showing α-SMA, collagen I and fibronectin expression in PaSCs; densitometry analyses of the blots is also shown. 1. MOCK; 2. NC; 3. Notch3 siRNA; 4. LTPA-conditioned medium; 5. LTPA-conditioned medium + Notch3 siRNA. *P < 0.05, **P < 0.01, and ***P < 0.001; Student’s t-test; n = 4. Bars represent mean ± SD. (TIFF 749 kb) [file 12885_2017_3957_MOESM1_ESM.tif]
